# Finding ways to carry on: stories of vulnerability in chronic illness

**DOI:** 10.1080/17482631.2020.1819635

**Published:** 2020-09-20

**Authors:** Oddgeir Synnes, Aud Jorun Orøy, Målfrid Råheim, Liv Bachmann, Else Mari Ruberg Ekra, Eva Gjengedal, Magnhild Høie, Else Jørgensen, Ragnhild Karen Astad Michaelsen, Hildegunn Sundal, Solfrid Vatne, Else Lykkeslet

**Affiliations:** a Centre of Diaconia and Professional Practice, VID Specialized University, Oslo, Norway; b Faculty of Health Sciences and Social Care, Molde University College, Molde, Norway; c Department of Global Public Health and Primary Care, University of Bergen, Bergen, Norway; d Department of Health and Nursing Sciences, University of Agder, Kristiansand, Norway

**Keywords:** Chronic illness, vulnerability, narrative analysis, life world, stories, phenomenology

## Abstract

**Purpose:** In this study, we explore the lived experiences of chronic illness in four groups of patients; children with asthma, adolescents with diabetes, young adults with depression, and adult patients with chronic, obstructive pulmonary disease (COPD). Persons living with chronic illness are often designated as vulnerable. This study builds on the assumption that being vulnerable belongs to being human, and that vulnerability also might entail strength and possibilities for growth.

**Methods:** A narrative analysis was undertaken to illuminate how experiences of vulnerability were narrated across the four patient groups, presenting four individual stories, one from each of the patient groups.

**Results/conclusion:** The stories illuminate how living with a chronic illness implies differing capabilities and capacities dependent on the specific condition. At the same time the stories point to how various abilities and challenges in living with chronic illness can be alleviated or seen as resources. Considered together, the stories underscore how ´finding ways to carry on´ in chronic illness requires interpretational work. By calling upon resources among significant others, in the surroundings and in oneself, the narrator can find ways of interpreting living with chronic illness that might open towards a hopeful future.

## Introduction

Chronic disease is the largest cause of death in the world (Yach et al., 2004), and noncommunicable chronic diseases (such as cardiovascular diseases, diabetes, chronic respiratory disease, chronic kidney disease, cancers), are one of the major health and development challenges of the 21st century, in terms of human suffering as well as socioeconomic burdens on society (Hajat & Stein, 2018; World Health Organization, 2014).

Persons living with chronic diseases are often designated as vulnerable (Gjengedal et al., 2013). This study builds on the general assumption that being vulnerable belongs to being human and is connected to being dependant on each other and our mortality as bodily beings (Vetlesen, 2001). Vulnerability is an invariable premise of life (Vetlesen, 2001). When chronic illness strikes us, we are reminded of our mortality. We are also more dependent on others, including professional helpers, and hence more vulnerable. Vulnerability often has a somewhat negative sense. This may put us in danger of overlooking the fact that living with illness may also contribute to personal growth and development (Carel, 2008, 2009; Malterud, n.d. ; Malterud & Solvang, 2005). Carel (2009) makes a distinction between objective and subjective vulnerability in relation to illness. She claims that there is no necessary relation between having a chronic illness and feelings of subjective vulnerability, even though the subject may objectively be more vulnerable.

Research on the phenomenon of vulnerability in healthcare points to several dimensions: Vulnerability is an existential phenomenon that belongs to the basic conditions of life, and it is a contextual phenomenon, which varies depending on the situation and the cultural context. It is also referred to in the research as a relational phenomenon; the relationship with others may increase and/or decrease the feeling of being vulnerable (Gjengedal et al., 2013).

In this study, we explore the lived experience of being chronically ill, in four groups of patients: children with asthma, adolescents with diabetes, young adults with depression and adult patients with chronic, obstructive pulmonary disease (COPD). These persons are all living with diseases with great impact on their lives.

To understand the process of living and coping with chronic illness or disability, several models and stage theories have been developed. Moos and Holahan (2007) developed a conceptual framework that considers chronic illness or disability an extended life crisis. The model describes adaptive tasks and coping skills and identifies primary determinants for adaptive coping. In our context, the model contributes to a wider understanding of chronic illness as a burden as well as a resource. Paterson (2001) developed a model concerning living with chronic illness, based on qualitative research. This model focuses on the shifting character of living with chronic illness, between foregrounding being sick and looking upon oneself as sick, or being healthy and looking upon oneself as healthy. Being a victim versus being the creator of one’s own circumstances relates to these shifts. In his famous book *The* *Illness Narratives*, Kleinman (1988) also directs our attention to shifts and fluctuations in the experiences of chronic illness, and the essential differences in the experiences related to the meanings ascribed to the illness by others, society and the sufferer. The meanings of illness intertwine with cultural and symbolic meanings inherent in the way illness is perceived and interpreted.

Telford et al. (2006) explored the terms *acceptance* and *denial* in their literature review; the concepts are sometimes used in old stage theories, with the aim of understanding the implications of using these concepts to categorize people’s responses to living with chronic illness. The authors stated that these notions may be a hindrance to hearing the unique stories of persons living with chronic illnesses and how they experience life with such illnesses.

Striving towards normality is an aspect of living with chronic illness that is highlighted in the research. The experiences of living normal lives despite chronic illnesses, or at least the informants’ wishes to live as normally as possible, are pointed to as essential (Gjengedal & Hanestad, 2003; Gloria & Acorn, 2000). Being normal is an identity work for people living with chronic illnesses (Heaton, 2015). Heaton (2015) explored how young adults used social comparisons in their experiences with chronic illnesses. By comparing and contrasting themselves to others and to their younger selves, the informants of this study created positive interpretations of their experiences. Ferguson and Walker (2014) found that adolescents living with chronic illness managed life by focusing on opportunities in a positive way. The informants showed a resistance to being seen as different and demonstrated an ability to reflect on their personal situations with optimism. Taylor et al. (2008) published a literature review to make recommendations for future research and clinical practice. Seven main themes were suggested: developing and maintaining friendship, being normal/getting on with life, understanding the importance of family, taking on a positive attitude towards treatment, having positive experiences in school, establishing good relationships with healthcare professionals and thinking about the future. The first one, developing and maintaining friendship, was the most important aspect. Nilsson et al. (2016) pointed out the importance of balance for people living with long-term illnesses. The balance is shown through striving for independence, having care that meets their needs, being together with others, being seen and understood in a network that works, choosing one’s own way and being able to see possibilities in life.

The research articles we have presented here do not explicitly address vulnerability; rather, they focus on people living with chronic illness. They show how patients strive to maintain their lives, and they point to important conditions related to their living well. An underlying theme is that people with chronic illnesses strive to maintain a normal life despite their illnesses. As already mentioned, Carel (2009) claimed that it is not necessarily inevitable that a relationship exists between having a chronic illness and the experience of vulnerability, even though a person with chronic illness may objectively be more vulnerable. In this current study, we wanted to more specifically focus on the connection between living with chronic illness and the phenomenon of vulnerability. The aim of the study is to explore the ways in which vulnerability expresses itself in the experiences of persons’ living with chronic illness. Insights from the study may be of importance to healthcare professionals who are responsible for the treatment and care of these patients.

## Methodology and methods

The research project is methodologically anchored in a phenomenological life world perspective and thus has a qualitative design (Bengtsson, 2006). Phenomenological research has gained rather substantial influence during recent decades on the research involving health professions, such as nursing, psychiatric nursing, physical therapy and occupational therapy. A possible reason is the closeness to practice through its in-depth explorations of the issues associated with the experiences of patients, clients, users and health workers. The themes addressed have great significance for clinical practice, and may therefore have substantial consequences for how health workers understand, meet and care for people with varying degrees of failing health.

In researching the lived experiences of vulnerability, we have been inspired by Paul Ricoeur´s phenomenological hermeneutics (1981). For Ricoeur, hermeneutics and phenomenology exist in a “mutual belonging” (1981, p. 101). Language is dependent on and refers to a phenomenological and pre-linguistic level, while the lived experience needs to be interpreted through language to be understood. This mutual dependency is clearly seen in Ricoeur´s work on narrative, where he argues that human life has a “pre-narrative quality” (1991), which is a practical understanding where we experience the world as more or less meaningful, and where we regard the various actors in the world to have motives; and where human actions are symbolically mediated through signs, rules and norms. This pre-narrative quality of life is the basis for our narrations of the world when we attempt to interpret experiences into a meaningful whole.

The present study aimed to investigate how a variety of patient groups narrated their experiences of chronic illness. How is human vulnerability influenced by illness and what significance does this have in a life world perspective?

### Data production

Four relevant participant groups were chosen: (1) children with asthma, aged 8–12; (2) adolescents with diabetes, aged 13–17; (3) young adults with depressive illnesses, aged 18–29; and (4) adult patients with COPD. The choice of the four groups of conditions, as well as variety in age were decided upon to give a broader perspective when trying to understand facets of chronic illness, both in somatic as well as mental illness and across different age groups.

Four participants from each group were included, and both genders were represented. Three qualitative research interviews were undertaken with each participant. Due to the aim of the project, to understand aspects of lived experience in chronic illness, we decided to do three qualitative interviews with each informant, to make sure that we got a rich and in-depth understanding of the informants´ everyday life over time.

The choice of four informants in each group was based on a pragmatic reasoning. The study´s aim of an in-depth investigation of each informants’ illness experience through three qualitative interviews represented a limitation to how many interviews that were feasible. The research team decided that four informants would give possibility for variation and width without the material getting too big. A total of 16 informants were interviewed three times.

A convenience sample was recruited. After getting permission to conduct the study in the hospital trust, we contacted the head of the actual ward or the outpatients’ clinic for help to recruit the participants after criteria given for participation. The head of the actual ward or outpatient clinics both presented the information letter, requested participation, and obtained informed consent from the patient or user/family. After the patient’s or user’s/family’s consent, they contacted the researcher to conduct the interview. The researchers repeated the terms of participation for the participants.

Most of the initial interviews took place when the participant was visiting a day clinic or admitted to the hospital. The next interview was done some weeks later. The interviews were semi-structured, and the participants were encouraged to talk about how they experienced being vulnerable, in a positive as well as a negative sense. In the study, we wanted to explore the ways in which vulnerability expressed itself in the patients’ experiences. The concept of vulnerability was not something that was stated in the interview guide. All the interviewers agreed upon beforehand not to focus explicitly on the concept of vulnerability, but to bring it up in the conversation if it felt relevant during the interview situation. In addition, we addressed their experience with illness and treatment, thoughts along the way, encounters with health personnel, transfers to the home, relations with family after coming home and thoughts about the future.

The researchers wrote a narrative based on the two previous interviews. This was presented to the patient in the third interview. In this third interview, the participant was given the opportunity to add nuances and elaborate on the story. All the interviews were recorded and transcribed, in total forty-eight interviews. Due to lack of time, three interviews were transcribed by a secretary.

### Narrative analysis

During the interviewing, the research team (the authors of this article) had multiple meetings discussing possible ways of analysing the material. The first author is an experienced narrative researcher. In the discussions, it was suggested that the material would lend itself to a narrative analysis. In the decision on a narrative analysis, we were inspired by Ricoeur´s phenomenological hermeneutics and his theory of how our experiences are interpreted and made intelligible through symbols and narratives. Narrative analysis is a diverse analytic tradition within qualitative research, which argues that people understand their situations and lives through stories (Josselson, 2011; Riessman, 2008). In qualitative health research, a narrative understanding of health has underscored how illness is a biographical as well as a biological phenomenon. In many instances, illness might imply a biographical rupture (Bury, 1982; Frank, 2013), as well as a need for a narrative reconstruction of the patient’s life story (Williams, 1984). However, not all conditions imply a biographical disruption, e.g., people who have been living with an illness their whole life. As Bury (2001) argues, stories of chronic illness can illuminate how people “seek to order experience in a temporal sequence, as the events of the illness unfold (its onset, diagnosis and treatment) and give expression to the changed relationship between body, self and society” (p. 278). Narrative research thus underscores looking at the whole account as a meaningful unit for analysis, instead of ordering the analysis around themes or categories across different interviews (Josselson, 2011; Riessman, 2008).

Reading through the different interviews, we found that the phenomenon of vulnerability, in many instances, best could be explored and illuminated by looking at the accounts of the participants as whole stories striving to create meaning and sense of their situations. Medical sociologist Arthur W. Frank (2012) asks; “How are people telling stories to discover who they are and to explore who they might become?” (p. 50). According to Frank, illness stories are vital capacities for an individual to *sustain a self that is under threat*. In light of this, we decided on the following analytic question when investigating vulnerability as both a resource and a restriction:

How do the participants sustain stories of themselves in vulnerable situations?

This overarching question required reading the interviews as a whole, and trying to understand how the different narrators gave their accounts in various ways in light of their situations. The research team then read the interviews, as well as literature on narrative analysis, and opted for five subsidiary questions that were both theoretically founded and based on what we found to be of importance in the interviews. These five questions all underscore crucial aspects of narrative meaning making that would help answering the overall research question (see Frank, 2010, 2012; Josselson, 2011; Riessman, 2008):

What are the significant events of the stories? (the ordering of the plot of stories are often centred around hallmark events)

How do the stories interpret the connection between present, past and future?

What are the central characters in the stories? (Stories are primarily character driven.)

In what settings are the stories played out? (Stories are always local, giving strong emphasis to location.)

What different voices of the self are found in the stories? (Stories are multivocal, displaying tensions between different versions of the self, as well as calling upon larger cultural plotlines.)

In the presentation of the findings, we decided on a purposive sampling and presented four stories, one from each of the patient groups. A narrative analysis looks for a holistic understanding, not representativeness. We saw that several of the informants’ stories could be presented, but in order to be able to give an in-depth understanding of the meaning making in the stories as a whole, we could only delve into a few of them. The four stories were chosen as examples of rich narrative meaning making of vulnerability in chronic illness without being exhaustive of the whole material. In the presentation of the four stories, we show how the stories both differ from, but also relate to each other, giving a complex understanding of vulnerability in chronic illness.

### Trustworthiness

According to Riessman (2008), narrative truths are always partial and incomplete. To make sure that studies are trustworthy, she advocates among others things, “reliance on detailed transcripts; attention to language, contexts of production […] acknowledgment of the dialogic nature of narrative; and […] a comparative approach—interpretation of similarities and differences among participants´ stories” (2008, p. 193). Furthermore, making explicit methodological decision as well as describing and discussing how interpretations were produced is part of the reflexivity of narrative research (2008, p. 195). The above aspects were all applied in the research process. When we decided upon the four stories, these were—together with the other stories—thoroughly discussed and read among the research team to consider how and under what conditions and contexts they were collected. Different possible interpretations were discussed, discarded, re-interpreted and altered. The analysis was always grounded in detailed and elaborate extracts from the interviews.

### Ethics

The four groups of participants varied in age and illnesses, and the ethical assessment was adapted accordingly. Within research ethics, children are referred to as a vulnerable group, and researching children requires extra care and awareness throughout the research process (Fossheim et al., 2013). In the sub-study with children with asthma, informed consent was obtained from the child’s parents. The children were given age-appropriate information about the project and were made aware that the parents have the right to know what they have said in the interviews. The parents were offered the opportunity to participate in the interview.

In the informant group of adolescents with diabetes, informed consent was obtained from the child’s parents and the child. Children under the age of 16 are legally not obliged to sign consent, but we wanted to give the children in this age group the opportunity to respect their participation. The young persons and the parents were informed that the parents did not have the right to become acquainted with all the information provided in the interview if the child did not want this (excluding information that the researcher believed should be passed on to parents if it dealt with conditions that have serious consequences for the young person’s life and health).

Regarding the informant group consisting of young adults with mental disorders, they can be particularly vulnerable in relation to other people, which means that sensitivity to the informants’ reactions during and after the interview is required. The duration and location of the interview must also be considered. The interviewers were psychiatric nurses experienced in working with people with mental disorders.

It was crucial that the children, youths and young adults wanted to participate and that they knew they could withdraw from the project at any time, without justification. The children’s and youths’ first-hand experience and competence make a valuable contribution towards gaining insight, the understanding of their situation and the significance of living with a chronic illness. At the same time, children and youths are entitled to protection and methodological adaptation. An attentive attitude of the informants’ reactions when telling about their experiences was crucial. Prior to the interview, a written agreement was made with the unit manager in terms of how the informants would be taken care of after the interviews, should it be needed. This was never raised as a need.

The last informant group consisted of patients with COPD, who were admitted to an intensive care unit. These patients are often in a poor period of their illness, and it was therefore crucial that it was medically justifiable to ask the patient to join the study. Participation was clarified by the responsible physician in the department in collaboration with the responsible nurse. Agreements were also made with the responsible nurse regarding follow-up after discharge if necessary. For this patient group, breathing problems are central, which was taken into account during the interview.

## Findings

In presenting the findings, we highlight one main theme that is essential for all four stories: “finding ways to carry on”. In the four stories, we get to hear how the narrator—by calling upon his or her own resources, significant others and the surroundings—can find a way of interpreting living with chronic illness in ways that open the possibility of a hopeful future. However, the stories also vary in their interpretations, and they show considerably differences in how to accommodate the difficulties of the illness in their ongoing narrations. Held together, the stories underscore how vulnerability, in addition to being closely connected to specific diseases, must be seen as contextual and dependent on available internal and external resources.

**Ida (23): Coming to terms with the past**. Ida grew up with a single mother. Her mother was struggling with severe mental illness and drug related problems. This affected Ida and she took on the role as an adult in her family *“from when I was a little girl actually.”*

In her teens, Ida became aware of the difference between her home situation and that of others. “It came to me especially in secondary school. I got more aware of the stressful situation and of what was happening back home. I had many thoughts about how life would have been if I had not been there anymore.”

She experienced a breakdown when she moved away from home to study:

The second year at the university college was the first time I really hit the wall. I believe triggering factors were failing the exam, not mastering my part-time job, and braking up with my boyfriend. Often I felt worn out, it was demanding, especially for my head with symptoms of migraine and a feeling of knot in my stomach.

Altogether, these problems resulted in a loss of motivation and a decision to end her studies and move back to her hometown.

A close relative understood that something was wrong when Ida isolated herself. The relative took hold of Ida’s situation and connected her with mental health services.

Through therapy, Ida has been able to see how her upbringing has affected her situation today. She has learned more about her own reactions and limitations. Talking to the mental health nurse has helped her be more open to herself and to people around her. She is now aware of the supportive persons in her life: *“I do think my family care about me. I do think they have cared previously as well, but I have not been able to receive it until now. I am more observant towards their support to me.”*

For Ida, not being seen by others has been a continually strain during her upbringing. “Why didn´t anybody see us? Why didn`t they help? I ask these questions myself.”

Also, later in her life, the lack of being seen has been central. Ida highlights this as a wound she brought into the relationship with her boyfriend and into her job situation at the time of the breakdown. This continues to be part of her present situation and is something she stresses: *“It is important to be seen, even if you are six or twenty years old.”*

Moving back to her home place, Ida was seen by a close relative and subsequently by the mental health nurse. Through these experiences and through therapy, Ida was able to realize the resources she had and has around her: her father´s family, a supportive grandparent, her new boyfriend and a few close friends.

In the interviews, Ida could now elaborate both positive and negative experiences with mental health problems and being vulnerable:

The positive is that you are getting to know yourself in a better way. Learning about your own limitations and learning to recognize them after a while. Learning to say “no” has been positive for my sake. A clearly negative way is that you are in a vulnerable situation. It is very heavy when it storms. You don`t know what to do or whom to talk with. You have a strong need of being seen, and you are in deep need of having someone to talk to. I feel I have been more observant in the way I struggle in the present, as well as in the past.

Despite reflections about the problems in the present and the past, Ida has now several goals in life, and about the future, she says the following: *“It seems brighter and brighter, having something to look forward to.”* Catching up with her studies again, she aims for a better life where she will be able to handle her everyday situation in a meaningful way: “I should be beyond the negative days by now. If I see one year back, I do see the positive way my situation has turned out. That helps quite a lot and gives me motivation to carry on.”

Reading Ida´s story, the plotline revolves around one main event: the breakdown and the subsequent reordering of her life through therapy and reaching a new outlook. In many ways, this story exemplifies illness as a biographical disruption (Bury, 1982) and the subsequent reconstruction of how to understand her life story (Bury, 2001). Even though Ida experienced a stressful upbringing, she understands through therapy how much her past has influenced her present situation. And in this narrative reconstruction, she also realizes the resources she has, especially her family and friends. A prominent feature of Ida´s story is her choice of wording when it comes to narrating her newfound understanding. She emphasizes the visual aspect of seeing and being seen. First, she has her own need to be seen by others, and secondly, she has a new realization of seeing and observing the resources around her. This makes way for a possible positive future. As such, it almost reads like a conversion narrative (I once was blind, but now I see). Despite the positive tone at the end of Ida´s story, tensions running through it are apparent, and the vulnerability is still very much present.

**Hans (12): Participating by one’s own means**. Hans (12) has quite a different story, which is not characterized by a big change. Rather, his vulnerability has been there his whole as he had asthma from birth: *“I do not know what it’s like to have a normal life.”* Hans uses the term *normal life* for a life without disease and wonders how that must be. Hans lives in a small village. His parents are divorced, and he lives with his mum; his dad lives far away.

Hans seems quite familiar with his situation, but his vulnerability appears in many episodes in his story. In his daily life, he often experiences shortness of breath, and his throat swells up. But he usually finds his own solutions to this, and during the interview, Hans presents us with several episodes that show how he manages his situation, such as the following: *“I was thirsty and my throat swelled up. I borrowed the toilet at the grocer´s shop and drank water from the tap*.”

His mother is the central character in his life and the one that takes care of everything (e.g., fixing things when he’s getting bad at night). “*I’m just trying to keep calm. I do not panic at all, so mom can do what she has to do.”* Twice he has been extremely ill and was hospitalized. It is boring to be in the hospital, but at the same time it is safe. “*I was almost dying; one time I was in a coma for a whole night. Yes, I have learned at least to appreciate that I am alive because twice I have been close to death. You may understand something more, just as I do: I am grateful that I live.”* He was serious when he said this. At the same time, he repeatedly said that he was not afraid during the interviews.

Hans can get sad and annoyed by people around him who do not understand what it’s like to have asthma. “People rarely think they are lucky because they are normal; they probably do not think that others may have diseases that can actually kill them.” His best friend lives across the road: “His father died of cancer, so he understands what it is to suffer through disease.” However, his best friend has now started in secondary school. Even though they still spend a lot of time together in their leisure time, Hans misses him in the schoolyard.

Hans likes to participate, especially playing football, although his breath limits him. “Sometimes I get into pain, almost unable to breathe at all. If there is pollen outside, then I am getting worse throughout the day. My skin itches and I become tired of coughing and get exhausted.” Gym can also be challenging at school. “The teacher does not really understand. I use to manage myself and know when I cannot participate in activities. When I express this, the teacher takes it as an excuse. It is not funny.” Hans can cope with the situation in swimming classes because he knows that he can swim. Hans is a good swimmer. When he talks about life at school, he says it is fine. However, hearing his story, certain tensions are apparent, and Hans admits it: “There is this special guy; he says I am cowardly because I am puffed.” He smiles a bit ironically and goes on: “Sometimes I’m nasty too—it’s good to fight back instead of just being teased.”

The story is characterized by a strong will and the ability to find solutions to specific challenges in everyday life. This ability and a predominantly positive outlook on life make Hans interpret his future in a positive way. He is fond of watching sport programmes on television, especially soccer, and he goes to soccer matches together with his grandfather. At the end of the interview, he suggests that this interest might be a possible future career: *“I want to work with something in connection to soccer, like a soccer commentator … I love to comment when things are not good.”*

Reading the interview of Hans as a whole, we see how the story displays vulnerability as both precariousness and strength. Despite the seriousness of his disease, Hans can call upon internal and external resources that give an overall positive outlook on his life and future. His mother is the central character in his story; she helps him and is there for him if he becomes ill. The fact that Hans´s close friend can understand him because of his own experience of vulnerability is important for the story. But, maybe the most salient feature is how Hans presents himself as a protagonist with a strong will and ability to find solutions to challenges in everyday life. Despite his illness and the experiences of being bullied and not being able to participate in many situations, he finds a way to stand up for himself and a way to imagine a future life in soccer where he can participate by his own means.

**Emily (16): Integrating illness in everyday life**. Emily’s illness story started when she was attending an exam at school. Suddenly, she felt very bad: *“My eyes simply shivered so much that the text turned into a big lump of letters.”* She felt thrown into a new and scary situation, a complete shock when she was diagnosed with diabetes (T1D). Both Emily and her father cried in the car on the way to hospital. *“I sort of broke down inside*”, she expressed. She was especially afraid of needles, which she knew well through observing her big brother, who also has diabetes. Nevertheless, Emily said that she felt supported from the very start of her journey with diabetes by her parents and not least by her brother: *“He understands, he is very caring … He is my role model*”, she explained. At the hospital, she was involved in the education programme and treatment. Even though her illness represented an existential breach in Emily’s life, she was ready to take on her “new” life shortly afterwards and tell others about it.

From early on, Emily was and still is much motivated by taking control. Having discipline with food, being cautious when physically active and regularly measuring the blood sugar status soon became part of her everyday habits. Her doctor encourages her to let go of control every now and then, pointing to the fact that it is not necessary to be *“best in class”* all the time. For Emily, however, the scary knowledge about the late effects, which is triggered by her big brother’s risk taking for the time being, holds her tight to her “diabetes schedule”. It costs a great deal to live such a disciplined life, which is a never-ending endeavour. She also actively opposes stigma related to this disease, namely that getting diabetes is one’s own fault. She simply states that the diabetes is part of who she has become: *“I will say that it also becomes a part of oneself; I cannot imagine a life without having diabetes now.”* Emily expresses an optimistic attitude towards living with her disease and leaves an impression of being quite mature for her age. Still, she also seems fragile in her huge efforts to take control.

The safe haven of home is Emily’s fundament for taking part in life outside home. It was very important for Emily to inform schoolmates and teachers about the diabetes herself, and so she did. At present, she feels that her schoolmates accept her and that they behave quite normally towards her again. Also, in other social settings, Emily prefer to inform them about potential embarrassing events, such as when the insulin pump beeps, when she needs to measure the level of blood sugar, and so on. Being open and in charge of the situation implies control.

One arena that represents a total freedom from the illness is the stable, where she feels integrated and has her own horse. A long-lasting dream came true when she was given the horse as a present from her parents after she got diabetes. The relationship with the horse means a lot to her: *“When I enter the stable, whistling at her, she responds immediately—the way she greets me, I do feel I mean a lot to her.”* The stable is also a place of acceptance, safety and true friendship. The other riders are well informed, and one older lady takes responsibility to look out for her. They make no fuss about it when precautions are taking place.

Emily is adamant that the disease has also given her experiences and a different view on life that can be resources. Recently, she has spent time in a kindergarten as part of an occupational-related activity organized from her school and discovered she was able to take a special role in relation to children with special needs: *“If I encounter a child in kindergarten who needs special adjustments … I think I`m able to put myself in that child`s situation …. I`m also struggling with something everyone is not able to understand.”* She reflects on her experiences from living with a chronic illness as an advantage in this respect, paving the way for a possible future: *“My life has taken a new direction because of the diabetes. I want to work with children, especially those with a handicap or chronic illnesses.”*

Emily´s story ends with highlighting the biographical disruption that has led to other possibilities, underscoring vulnerability as a potential. While Emily is steadfast in her conviction that she will be able to live well with her diabetes and be able to create a meaningful future despite the challenges, her story opens with a rupture that is portrayed as devastating, both for herself and her family. At the same time, getting diabetes was not something that Emily and her family were totally new to. Having an older brother who had already had the same issues, Emily could call upon his support as well as the rest of the family. For Emily, her life revolves around the arenas of home, the stable and school. The support from home gives her strength to be open and integrate diabetes into her everyday life elsewhere too. This story has several aspects that can be linked to what Bury (2001) calls narratives of normalization in chronic illness: “‘Normal life’ is redesignated as containing the illness, and being open about it” (p. 272). At the same time, this story has similarities with Hans´s story, where vulnerability can lead to new perspectives and openings towards possible futures.

**Astrid (72): Struggling to accept a changed life**. Astrid is a woman in her early 70s who is married and has two children and several grandchildren. The family means a lot to her. As a person, she is both social and independent. Among her many interests, she loves hiking in the mountains, but the illness has put an end to that.

Her COPD has developed within a 20-year period, but Astrid has never used the term *COPD* to refer to her condition. She was previously a smoker but stopped due to the illness 15 years ago. She does not relate the illness to smoking. She was born into a family of asthmatics and says she has asthma, not COPD, because she associates the correct diagnosis with shame. She also suggests that the disease can be genetically conditioned. Despite the fact that her family knows about her COPD, Astrid herself wants to decide when she is in need of professional help.

During the interviews, Astrid mentions three episodes of deterioration. The first happened many years ago when she was on a trip with some friends. A humid indoor climate and a strong cold triggered a serious situation. One morning she woke up with great breathing problems. Her condition deteriorated quickly, and she needed medical help. When the doctor tried to give her oxygen treatment through a mask, she panicked. For the first time in her life, she was hospitalized for her COPD. After this event, she lived almost “normally” for many years. Even though she does not endeavour to take the toughest hikes, she also does not experience the disease as an obstacle, and she does not regard herself as chronically ill.

Some years later, the second dramatic event occurred. Astrid explains that she was doing some renovation. “Suddenly I could not breathe, and thought I would die. I completely fell away and do not remember anything before I woke up at the hospital.” She was hospitalized for 14 days. She says it was a terrible experience when she was put on a ventilator and was not able to talk.

The last serious incident happened a short time ago. Astrid knew she was sick, but did not want to go to the physician. She says that something happens with her thought process when the breathing problem arises. Even though she has knowledge and experience with COPD, time after time she asks for help too late. This time was no exception. Again she was taken to the hospital, and this time she was also put on a ventilator. She describes the experience as something she would not want her worst enemy to have. Time stopped. She felt she was being stifled and everyone wanted to hurt her. She was worth nothing, and no one wanted to help her. Not being able to speak was also terrible. *“I thought they would ‘kill’ me”*, Astrid says.

After this experience, she has come to the conclusion that she must apply for help earlier. Now she experiences the disease more as a threat than before and has accepted the idea that her spouse should take greater responsibility for contacting the healthcare system when he understands that it is necessary. Because she does not have the classic symptoms, such as cough and mucus, she must be aware of other symptoms, such as losing appetite and feeling tired and miserable. Still, she feels “uncomfortable” when she goes to the physician, and he cannot find objective signs, such as increased CRP or high temperature.

After the last hospitalization, Astrid’s condition has deteriorated further. Now she is dependent on oxygen at home and a mask treatment at night. She also lost her driver’s licence, which was a big blow to her because she became more dependent on others. Accepting the new situation has been challenging, but she is solution oriented and motivated to cope with the situation. Today, she and her husband take a taxi when they need to get around and feel both freedom and comfort with that. She can also move outside with a walker and oxygen without feeling ashamed—even though this took time and considerable courage. Astrid has hopes for the future and says she will consider making changes to their house or moving to an apartment if necessary for her health.

The story of Astrid revolves around how to come to terms with a changed life. Several researchers have emphasized how many stories of illness are stories of morality and about helping to maintain self-worth and doing what is right and virtuous in a given situation (see Bury, 2001; Frank, 1997; Williams, 2002). For Astrid, her story of coming to terms with her illness is centred around questions of morality and interpreting the illness as something that cannot be blamed on her and her previous life as a smoker. Her ideals of being independent and managing her illness had to be renegotiated, and she can now still feel independent, though in altered ways. She can no longer drive a car, but she can take a taxi with her husband. She can no longer take walks in the mountains, but she can move freely and without shame outside with a walker and an oxygen tank. And she can rely more on the support of her husband instead of always taking control of everything herself.

## Discussion and reflection

The four stories are examples of living with chronic illness in everyday life. These illnesses are characterized by acute, unstable and stable phases. We asked the overall research question: *How do the participants sustain stories of themselves in vulnerable situations?* The analysis of the four stories has shown how living with chronic illness requires work and effort in mundane practical everyday activities, in encounters with significant others and their surroundings, in exchanges with the healthcare system and in the management of their illness. As such, the stories underscore how vulnerability is always contextual and relational (Gjengedal et al., 2013). However, the analysis has also highlighted the importance of “holding their own” (Frank, 2015) in a vulnerable situation because the chronically ill require interpretational work. Understanding how the four storytellers found *ways to carry on* despite their illnesses required a narrative understanding. It is by hearing how the storytellers interpret the main events in light of their previous life stories, how they interpret living with the disease, and how they draw on the available resources in and around themselves that we can understand their vulnerability, as well as how and by what means they can find a way towards a possible future.

The participants’ stories visualize different ways of striving “to carry on”. Three of them have experienced illness as breakdowns in their lives, and the stories subsequently revolve around making sense of new situations. For instance, Emily was 14 years old when she got the diagnosis of diabetes. Her striving is directed towards controlling her life with diabetes and making it a part of her everyday life. The scary knowledge of the late effects triggered her to be disciplined about food, to be cautious when physically active, to regularly measure her blood sugar status, to take her medication and so on. However, the illness has also provided a newfound resource in how she understands more about life and has opened up the possibilities toward a future career where her experiences can be an asset in helping other people.

In contrast with the other narrators, Ida strives to come to terms with the past just as much as the present. She took on an adult´s role in the family and needs help from professionals to see how her childhood has influenced her life. She had to learn about her own limitations—to say no, to trust family and healthcare professionals, to be aware of and be able to receive care. Her experiences of not being seen by others has been and is a continual strain. For her, professional help is necessary to carry on. Astrid initially denied the diagnosis of COPD. After being diagnosed, she stopped smoking, took her medication and continued a “normal” life. Astrid is struggling to accept a changed life and drawing upon resources outside of her experience. As the COPD worsened with episodes of acute breathing problems, she gradually accepted the illness. Her struggle is related to her wish of being healthy and independent on the one hand, and losing control because of the unpredictability of the illness on the other. In contrast to these three narratives, Hans has no experience of living without asthma; he takes his illness for granted. His striving is related to adapting to the challenging situations he faces in his everyday life due to the problems with his breathing and his throat swelling up. But in his story, the striving to carry on is perhaps even more related to the tension between those who do not understand the severity of his breathing problems and those who understand and support him.

Despite the differences, the four stories display common traits regarding vulnerability. Bury (2001) argues that narratives of chronic illness are played out in the interplay between *contingent narratives* (the interpretation of the illness in the everyday life), *moral narratives* (establishing the moral status of the individual) and *core narratives* (available cultural narrative resources as tragic and heroic narratives). The stories of Ida, Hans, Emily and Astrid all display elements from these categories that they use to sustain themselves. By and large, the four stories can be interpreted in light of the cultural hegemonic narrative (core narrative) of heroic stories of illness when they interpret the illness in their everyday life. In various ways, these are stories where the storyteller displays significant strength and agency and refuses to be victimized and to let the illness define his or her life course, or as the research of Paterson (2001) underlined, one strives to be the creator of one´s own circumstances. Furthermore, they all illustrate a strong will to continue living—and living well—despite the illnesses in their everyday lives by accommodating the illnesses into their situations in the here and now. But the stories are not just rehearsals of an available narrative plotline of heroic illness. They are also *moral* stories of individual narrators who display newfound strengths and outlooks on life. As such, these stories can also be seen as variants of *quest stories* where the illness can give rise to a newfound understanding of life and appreciation of what one still has, as well as new opportunities (Frank, 2013). Still, despite the fact that the narrators, in their various ways, manage to find a positive outlook, we also notice the fragility that is displayed in all the stories, reminding us of the vulnerability in their lives and of how a ´narrative truth´ is always one version among many possible ones.

In the following, we deepen the understanding of these illness stories from a phenomenological perspective intertwined with a narrative understanding of lived experience. Living with chronic illness makes various changes in human beings’ ability to be or to exist in their physical and social surroundings. Heidegger (1927/1962) describes human existence not just as “being-in-the-world”, but as “being able to be in the world”. “Being able to be in the world” means that openness to becoming capable of this or that (e.g., being a carpenter, a teacher, a football player or a journalist or assuming different social roles) characterizes human existence, and may open up opportunities for understanding that a person can become whatever he or she wants (Carel, 2009). Carel (2009) criticized this view of human existence because it does not include physical and psychological limitations in health. When a person is chronically or mentally ill, the person’s ability to be in the world is radically changed and requires curtailing activities, as in the four stories described in this article. The participants’ stories show various limitations related to being able to be, and in some situations, they also became unable to be what they wanted. A glimpse into Hans’s story visualizes his will to participate in a “normal” life by his own means. His story is solution and future oriented. Despite his dependency on others and the graveness of his previous illness episodes, he tells about an inner strength to carry on. He is aware of the illness limitations and expresses the sad notion that he cannot fulfill his dream of becoming a professional football player. Still, he has future prospects that hold great opportunities. He can certainly become a soccer commentator. This is Hans’ way of imagining a future of being able to be in the world. It is also a way of holding his own.

Carel (2009) wanted to expand Heidegger’s definition in two ways. First, the notion of “being able to be in the world” must be broadened to include radically differing abilities. For instance, Emily imagines an ability to carry on in a new way. She expresses a strong wish for working as a professional with children with special needs in the future. Because of her illness experiences, she feels able “to put herself” into those children’s situation and, in such a way, care for others. Astrid also sees that she has different abilities that can allow her to continue her life. Despite restrictions from being ill, she has opened up for new possibilities to be able to be in her rapidly changing situation. Now she has accepted the idea of taking a taxi when she needs to get around, which was out of the question for her some time ago. She also moves outside with a walker and oxygen, without feeling ashamed, which is also quite a new experience. Furthermore, she has hope for a future when she considers moving to a functional apartment to handle her limitations.

Secondly, the “inability to be” needs to be recognized as a way of being-in-the-world. One example is Ida’s descriptions of the positive and negative experiences in living with mental health problems. During therapy, she has learned to know herself and how to live with her vulnerability. This has been an ongoing process. Her life seems brighter now, and she says that she has something to look forward to.

Being inable to be is always linked to being in a new way. Our participants’ illness narratives visualize persons who live with their illnesses and carry on in new ways. As mentioned earlier, carrying on is not an independent or context-free concept. Through these narratives, we may see striving and sometimes struggling, indeed, to continue living meaningful lives. The illness stories are examples of people being vulnerable in finding themselves in a context of inability. This position challenges persons living with chronic illnesses to transform inability to the ability to be in the world.

Expanding Heidegger’s definition of being in the world to being able to be in the world seems to include all persons of all ages, as well as persons living with chronic illnesses, such as the participants in our study who all have different conditions with different life prospects.

## Conclusion and clinical implications

The main purpose of this study was to explore the ways in which vulnerability expresses itself in patients’ stories about their lived experiences of chronic illness. Our view on vulnerability is built on the general assumption that vulnerability belongs to being human, and that vulnerability is part of the invariable premises of life. In some situations, human beings’ vulnerability is more than ordinary, as with those living with chronic illness. The vulnerability expressed in the narratives presented here seems profoundly linked to the participants’ continuous striving to carry on.

Listening to the stories the patients tell paves the way for healthcare professionals, family members, and others to share inside views of illness experiences, which may truly be helpful in helping patients to find their own ways of carrying on with their lives.
